# Efficacy of intravitreal dexamethasone implant in patients with Vogt–Koyanagi–Harada Disease and bilateral panuveitis: Two case reports

**DOI:** 10.1097/MD.0000000000027394

**Published:** 2021-10-08

**Authors:** Po-Lin Chen, San-Ni Chen

**Affiliations:** a Department of Ophthalmology, Changhua Christian Hospital, Changhua, Taiwan; b Department of Ophthalmology, Eye Center, China Medical University Hospital, Taichung, Taiwan; c Department of Ophthalmology, China Medical University, Taichung, Taiwan.

**Keywords:** dexamethasone intravitreal implant, ozurdex, panuveitis, Vogt–Koyanagi–Harada

## Abstract

**Introduction:**

Vogt–Koyanagi–Harada (VKH) disease is a multisystemic disorder characterized by intraocular inflammation associated with serous retinal detachment, optic disc edema, uveitis, and vitritis, and is often associated with neurologic and cutaneous manifestations. Diagnosis can be assisted by fluorescein angiography and optical coherence tomography that can help evaluate changes in the retina. Therapy relies mainly on the use of corticosteroids, administrated through oral or intravenous high-dose pulses, and immunosuppressants. The purpose of our study was to assess the outcome of VKH disease with bilateral panuveitis treated with dexamethasone intravitreal implant.

**Patient concerns:**

Two patients without underlying disease had severe vision deterioration, eye pain, following flu-like symptoms.

**Diagnosis:**

At initial diagnosis, macular edema and sub-retinal fluid lobulated accumulation were noted under SD-OCT exam. FAG revealed multiple pinpoint leakage around macula and pooling of dye within sub-retinal space.

**Interventions:**

All two patients received intravenous pulse methylprednisolone at the diagnosis, followed by oral prednisolone and cyclosporine. One patient received bilateral eye dexamethasone intravitreal implant two weeks after diagnosis, while the other received left eye dexamethasone intravitreal implant at the time of diagnosis.

**Outcomes:**

Vision and macular structure recovered more rapidly after receiving dexamethasone implants in the short-term follow-up. All macular structures recovered to normal, and vision recovered to 20/20 in both eyes. One patient, receiving bilateral dexamethasone implant, didn’t relapse during the 13-month follow-up; the other, receiving left eye dexamethasone implant, didn’t relapse during the 6-month follow-up. None of them required intravenous high-dose steroids again.

**Conclusion:**

VKH disease is a multisystemic disorder; intravenous pulse steroid therapy and oral prednisolone can control systemic inflammation. In addition to systemic prednisolone treatment of VKH disease in the acute phase, dexamethasone implants can enhance short-term and long-term control of intraocular anti-inflammation.

## 1 Introduction

Vogt–Koyanagi–Harada (VKH) disease is a multisystemic autoimmune disorder, mediated by T cells that target melanocytes, and is associated with neurologic, cutaneous, and granulomatous panuveitis manifestations. It can be classified clinically into four different phases: prodromal, acute uveitic, convalescent, and recurrent or chronic stage.^[1,2]^

In the prodromal phase, the patient presents with flu-like symptoms for a few days to weeks. Most of the patients seek medical help in the acute uveitis phase due to acute blurring of vision in both eyes. Diffuse choroiditis and breakdown of retinal pigment epithelium (RPE), leading to focal exudative retinal detachment and sub-retinal fluid (SRF) accumulation. Vitritis and anterior granulomatous uveitis also occurred.^[1,2]^

Spectral domain-optical coherence tomography (SD-OCT) presents with multifocal accumulation of SRF. Fluorescein angiography (FAG) can present the breakdown of RPE; multiple hyperfluorescent leaking dots (pinpoint leakage) and hyperfluorescence pooling within sub-retinal space (pooling of dye) are typical findings.^[1,2]^

To prevent disease progression to sunset glow fundus, we need to control choroiditis, panuveitis and other multisystemic inflammation. The standard therapy consists of high-dose intravenous pulse therapy (1 g/day of methylprednisolone) for three to five days, followed by oral prednisolone, which was tapered within six months.^[1,3]^ However, in some patients with a disease of greater severity, systemic therapy cannot fully control the inflammation, while sub-tenon and intravitreal steroids provide a much better bioavailability.^[4–10]^ In this study, we demonstrate two cases of bilateral panuveitis with VKH disease successfully controlled by dexamethasone intravitreal implants (Ozurdex, Allergan).

## 2 Materials and methods

In this case report, four eyes of two patients were enrolled in this clinical research. Standard pulse therapy with methylprednisolone was arranged at the diagnosis. In case 1, both eyes received bilateral intravitreal dexamethasone implants at two weeks due to the slow response of intravenous methylprednisolone. In case 2, only the left eye received an intravitreal dexamethasone implant because of the worse BCVA. All the procedures were performed by one well-experienced ophthalmologist (SN Chen), and all the methods were carried out in accordance with relevant guidelines and regulations. This study was conducted under the approval number 210117 of the Ethical Committee of the Changhua Christian Hospital and the informed consents that clarified the permission for undergoing the surgery had been obtained from these two patients. All the medical records were collected since the diagnosis was made and subsequent 13 months (case 1) and six months (case 2) follow-up.

### 2.1 Case 1

**Figure 1:**
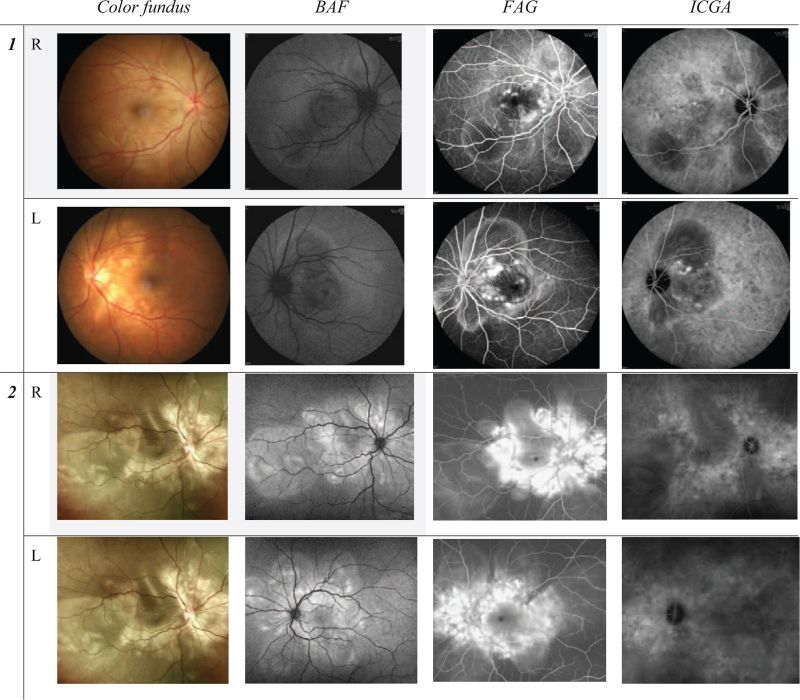
Ocular findings at initial first visit in the first (top and second rows) and second patient (third and bottom rows). Color fundus showed multiple loculated serous detachment at posterior pole in both eyes. Blue light fundus autofluorescence (BAF) showed hypo- and hyper- autofluorescence at the area of serous retinal detachment. Fluorescein angiography (FAG) showed multiple pin-point leakage with dye pooling. Indocyanine green angiography (ICGA) showed hypo-fluorescent circular area corresponding to the serous retinal detachment, along with multiple hyper and hypo-fluorescent dots.

A 40-year-old man visited our hospital due to recent bilateral vision loss, following flu-like symptoms of fever, headache, and neck soreness. The patient presented with bilateral acute visual impairment with best-corrected visual acuity (BCVA) of 2/200. Bilateral macular edema and peri-arcuate retinal whitening were also found. SD-OCT revealed bilateral macular edema and SRF lobulated distribution. **(**Table 1, ***Day 0*)** Fundus autofluorescence (FAF) showed hypo-autofluorescence in the macula. FAG showed bilateral multiple hyper-fluorescent pinpoint leakage around the macula, and large placoid areas of hyper-fluorescence, pooling within sub-retinal space. In indocyanine green angiography (ICGA), multiple hypo-fluorescent dark dots are located at the posterior pole and remain hypo-fluorescent until the late phase. **(**Fig. 1, ***Case 1*)**.

**Table 1:**
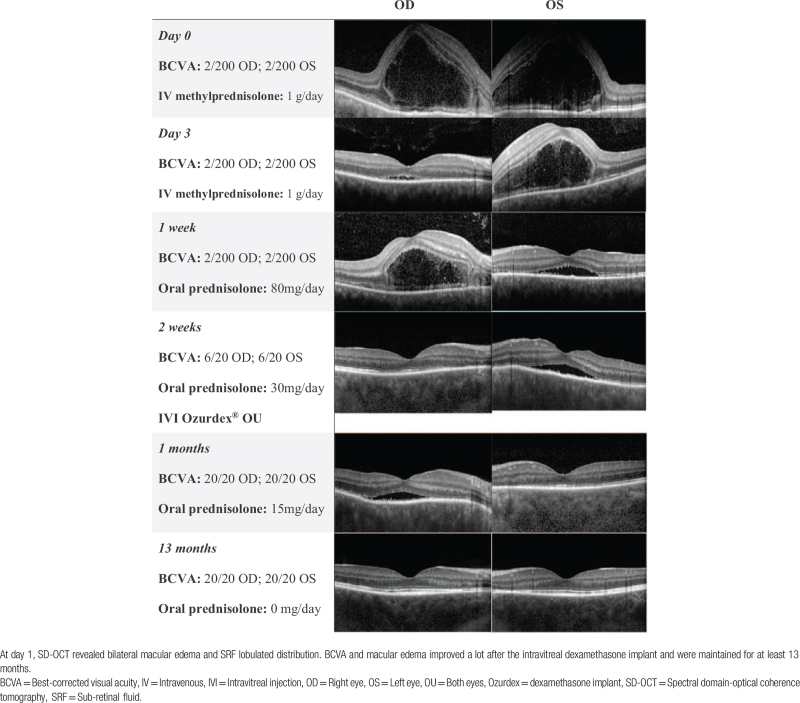
SD-OCT manifestation of Case 1.

After the diagnosis of VKH disease had been made, we arranged pulse therapy with methylprednisolone (1 g/day for 3 days), followed by oral prednisone (80 mg/day) and oral cyclosporine (300 mg/day). One week later, there were improvements in bilateral macular edema and BCVA; thus, oral prednisolone was tapered down (40 mg/day) and substituted with cyclosporine (300 mg/day). However, SRF persisted without further resolution. Because the patient wanted a rapid recovery, intravitreal injection with sustained release dexamethasone implant (Ozurdex, Allergan) was designed to achieve penetrative control of inflammation (Table 1).

After the intravitreal Ozurdex implantation, BCVA and macular edema dramatically improved. One week after the intravitreal Ozurdex, no SRF was noted; three weeks after the intravitreal Ozurdex, BCVA returned to 20/20 in both eyes. Timolol 0.5% eye drops were given to prevent the ongoing progression of ocular hypertension. Oral prednisolone was rapidly tapered down to low-dose steroids (5 mg/day) within two months after the Ozurdex injection and was tapped off at four months after the Ozurdex injection. His final BCVA was 20/20 and SD-OCT maintained no macular edema for more than a year (Table 1).

### 2.2 Case 2

A 33-year-old man visited our hospital due to sudden bilateral vision loss and bilateral eye pain for three days. The BCVA showed 4/20 in his right eye and 2/20 in his left eye. Fundus exam, SD-OCT, FAG, FAF, and ICGA showed the signs of VKH disease. **(**Fig. 1, ***Case 2*)**.

After the diagnosis of VKH disease, the patient received pulse therapy with methylprednisolone (1 g/day) for three days followed by oral prednisolone (80 mg/day). He had received an intravitreal injection of Ozurdex in the left eye due to the poorer BCVA on the same day the diagnosis was confirmed. (Table 2) Faster reduction of SRF and visual recovery was noted in the left eye in subsequent weeks. Steroid-induced glaucoma was observed in the left eye two weeks later, and Timolol 0.5% eye drops were treated to avoid ocular hypertension. Sub-tenon injection with triamcinolone was administered in both eyes due to poor reabsorption of SRF and slow BCVA improvement after two weeks of diagnosis. Two weeks after a sub-tenon injection with triamcinolone, SRF disappeared and BCVA improved to 20/20 in both eyes. Oral prednisolone was tapered down to low-dose steroids (5 mg/day) within two months and was tapered off at four months. No recurrent disease activity was noted during the six-month period.

**Table 2:**
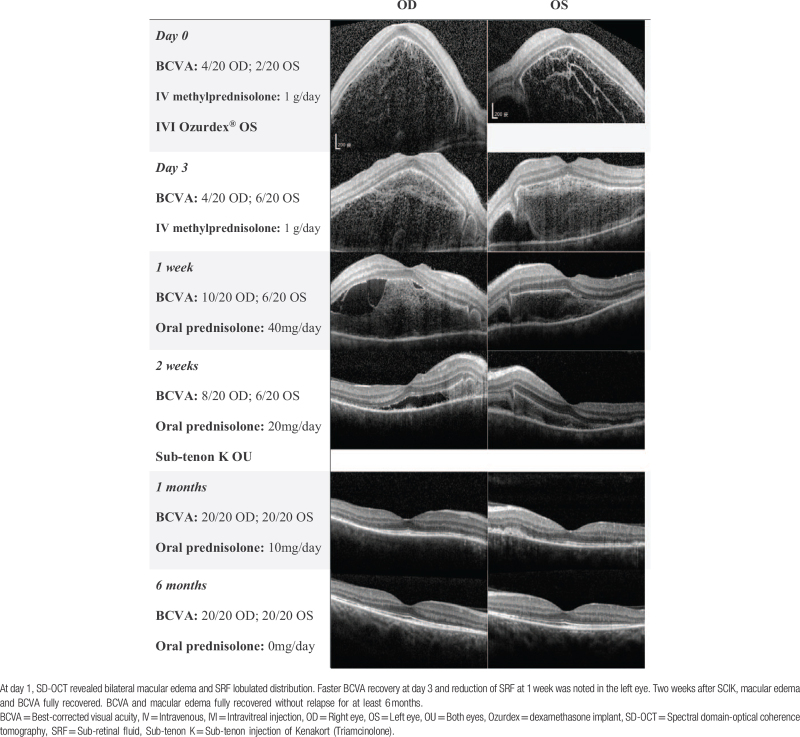
SD-OCT manifestation of Case 2.

## 3 Discussion

Corticosteroids are well-known for their anti-inflammatory effects, which can inhibit phospholipase A2 induction through lipocortin production, and further downregulate the production of many interleukins, prostaglandin, and tumor necrosis factor.^[5,11]^ By means of anti-inflammatory effects, corticosteroids have been applied to the vast majority of inflammatory diseases via several forms and routes. The treatment modality of VKH disease is primarily early intravenous pulse therapy, and is maintained by oral prednisone for months.^[1,3]^

Sub-tenon triamcinolone has been previously reported being effectively treated for uveitis, such as Behcet's disease, VKH disease, sympathetic ophthalmia and white dot syndromes, as well as for the acute phase and the recurrence of VKH disease.^[5,6]^ Recently, sub-tenon triamcinolone or intravitreal injection of dexamethasone implants together with systemic corticosteroids has been reported being effective in treating VKH disease which was refractory to systemic therapy.^[5,8]^ Other reports also show that local therapy alone may be effective in the treatment of VKH disease.^[6,10]^

In our first case, we performed intravitreal injection with an Ozurdex implant to prevent SRF re-accumulation and to adequately control the inflammation. With the help of the slow-release dexamethasone implant, systemic prednisolone is tapered at a more rapid rate than routinely done and can maintain without recurrence for more than one year. Oral prednisolone was tapered down to low-dose steroids within two months and stopped within four months after the treatment of Ozurdex without recurrence, unlike the previous study that suggested maintaining oral prednisolone for more than six months.^[12]^

In case 2, we observed a more rapid resolution of SRF and a faster speed of visual recovery in the eye receiving dexamethasone implant, which was a worse condition at initial presentation. Although the visual acuity is the same in both eyes at the final follow-up, rapid visual recovery can help patients returning back to a normal life in a shorter period of time. Sub-tenon triamcinolone has an effective anti-inflammatory impact on choroidal inflammation; two weeks after the treatment of triamcinolone, SRF is completely reabsorbed.

Intravitreal injection of dexamethasone implants can effectively control intraocular inflammation without the systemic steroid side effects, though local side effects, like steroid-induced cataract and steroid-induced glaucoma, and the high cost, are still needed to be considered.^[13]^

## 4 Conclusions

VKH disease is a multisystemic disorder; therefore, systemic steroid treatments, like pulse therapy and oral prednisolone, are generally served as first-line therapies. However, local therapy with intravitreal dexamethasone implant can provide a faster visual recovery and a faster tapering of systemic therapy and should be considered in patients with severe eye inflammation and patients who cannot tolerate long-term systemic steroid side effects.

### 4.1 Limitations

There are some limitations in this study. First, this is an observational study, and we cannot isolate the effect of intravitreal dexamethasone implants without the assistance of pulse therapy with methylprednisolone, sub-tenon injection with triamcinolone, oral prednisolone and other immunomodulators. Second, the case numbers are small. Further prospective studies with a larger case number are necessary to validate our conclusions.

## Author contributions

**Conceptualization:** Po-Lin Chen, San-Ni Chen.

**Resources:** San-Ni Chen.

**Supervision:** San-Ni Chen.

**Validation:** Po-Lin Chen, San-Ni Chen.

**Visualization:** Po-Lin Chen, San-Ni Chen.

**Writing – original draft:** Po-Lin Chen.

**Writing – review & editing:** Po-Lin Chen, San-Ni Chen.
